# A Case of Posterior Cortical Atrophy Presenting with Mood and Psychotic Symptoms

**DOI:** 10.1155/2024/2220082

**Published:** 2024-02-08

**Authors:** Tremearne Hotz, Manu Sharma, Bharat Narapareddy

**Affiliations:** Department of Psychiatry, Institute of Living, 400 Washington Street, Hartford 06114, CT, USA

## Abstract

Posterior cortical atrophy (PCA) is a rare neurodegenerative disorder characterized by predominant visual deficits due to its atrophy of the occipital lobes. Patients typically have preserved cognitive function during the early stages, making diagnosis more difficult when compared to other neurocognitive disorders. In this case, the patient presented predominantly with mood symptoms, delusions, and visual hallucinations. The disease course began 5 years ago with anxiety and insomnia. It developed into depressive symptoms including two suicide attempts (SAs), paranoia, and hallucinations. The diagnosis was eventually reached utilizing a thorough clinical exam, neuropsychological testing, MRI, positron emission tomography (PET), and dopamine transporter (DAT) scans. We conclude that mood or psychotic symptoms that emerge, escalate, or change dramatically at later ages merit further workup to evaluate for underlying neurodegenerative disorders.

## 1. Introduction

The majority of neurological disorders have significant psychiatric dimensions and vice versa. This overlap in presentations can often lead to misdiagnosis and in turn to inappropriate treatment and delay in receiving an accurate diagnosis [1–5].

Posterior cortical atrophy (PCA), a subtype of Alzheimer's disease (AD), has been shown to be one such neuropsychiatric disorder, often taking long periods to diagnose [6] and occasionally appearing primarily with affective complaints [7, 8]. PCA's predominance of atrophy in the occipital lobes can spare the patients memory, and instead display affective and visual symptoms. This makes misdiagnosis with mood disorders or other neurological disorders like Lewy body dementia (LBD) a major challenge. Here we present one such case, in which a patient was initially diagnosed at an outside hospital with major depression and general anxiety disorder symptoms. Upon admission to our inpatient facility, the patient exhibited considerable paranoia. Given his presentation along with collateral information, our team strongly suspected an underlying neurodegenerative disorder. Given the patient's symptoms of parkinsonism, possible visual hallucinations, and fluctuating cognition, LBD was considered on the differential before the final diagnosis of PCA was made.

## 2. Case Presentation

A 65-year-old man with a past medical history of generalized anxiety disorder (GAD) and major depressive disorder (MDD) was brought to the emergency department by his daughter after exhibiting paranoid and bizarre behavior for 6 weeks, culminating in the patient attempting to overdose on his medications that morning. When the patient informed his daughter of his suicide attempt (SA), she brought him to the hospital for further evaluation. The patient reported taking 10 pills of 50 mg trazodone and 10 pills of 10 mg paroxetine in an effort to kill himself.

Per the patient, he had been experiencing escalating depression and suicidal ideation (SI) for 2 months prior to this presentation. The patient associated his depression with extreme distress due to rosacea affecting his appearance as well as concern about the air quality and mold in his house. The patient reports waking up frequently in the middle of the night due to concerns of his rosacea. Notably, 2 days prior, the patient presented at a different emergency department for anxiety and depression without SI, and was discharged without admission. Since that time the patient's depression had escalated into SI.

Four weeks prior to presentation, the patient attempted to commit suicide and was hospitalized at an outside psychiatric facility for 1 week. The patient reportedly stepped in front of and was hit by a moving vehicle going 40 mph. At that time, the patient sustained a small subdural hematoma with no other medical complications. The patient was discharged on clonazepam 0.5 mg daily, trazodone 50 mg nightly, and escitalopram 10 mg daily. Notably, the patient was not prescribed an antipsychotic during or after this hospitalization as no symptoms were identified as psychotic in nature.

Per the patient's daughter, the patient had begun to act bizarrely for the past 6 months. This included paranoia that he could see mold and dust mites in the bathroom and for this reason refused to touch anything in the house. The patient was also concerned that people were hacking his cellphone and that the police would arrest him for a minor car accident he was in. The patient's daughter also reported that the patient had not been eating nor sleeping, and had new difficulty completing ADLs including showering, taking medications, and eating. Additionally, the patient was concerned that his memory had been declining due to issues with attention and word finding.

The patient has experienced issues with anxiety and insomnia since he retired 5 years ago, but had not received treatment until his hospitalization. He denied issues with depression until 2 months prior.

Upon presentation, the patient was found to be medically stable, alert, and oriented ×4, with a linear thought process and depressed affect congruent with his mood. The patient was cooperative with conversation and agreed to be admitted voluntarily into inpatient psychiatric care. Initially, while in the ED, the patient was restarted on his home regimen of paroxetine 5 mg daily, mirtazapine 10 mg nightly, losartan 100 mg daily, and trazodone 50 mg. Upon admission, trazodone and paroxetine were discontinued to reduce anticholinergic burden.

In the beginning of his admission, the patient became concerned that his phone had been tapped overnight and that staff was stealing his money and monitoring his movements with hidden cameras. Quetiapine was started and titrated up to 50 mg nightly to target psychotic symptoms, with the plan to increase to a therapeutic dose. After 2 days quetiapine was discontinued in favor of olanzapine, in order to more effectively target the patient's psychotic symptoms. Olanzapine was subsequently increased to 10 mg daily.

After the patient's symptoms did not remit for a week, an MRI and neuropsychological evaluation were completed to evaluate for a neurocognitive disorder. MRI showed mild generalized parenchymal volume loss and a few scattered nonspecific T2 hyperintense foci in the subcortical and periventricular white matter. Neuropsychological evaluation revealed deficits in executive functioning, visual construction, confrontation naming, learning, and memory, as well as poor insight into these difficulties. This pattern of weaknesses was suggestive of cortical involvement.

**Figure 1:**
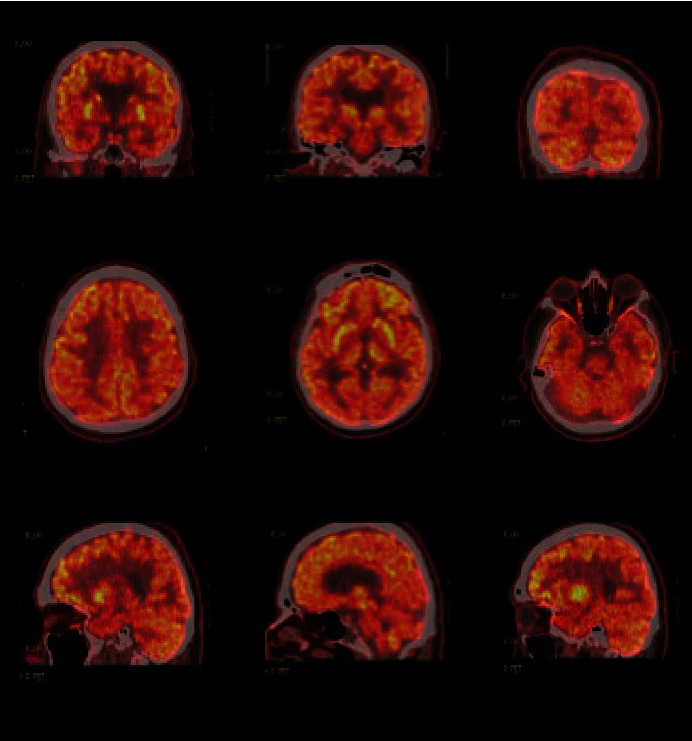
Fluorodeoxyglucose-positron emission tomography (FDG-PET) images of the patient demonstrating hypometabolism of the occipital and temporal lobes. Additionally, demonstrating the cingulate island sign (CIS): spared normal level of metabolism in the cingulate gyrus.

Given the suspicion for a neurodegenerative disorder, a fluorodeoxyglucose-positron emission tomography (FDG-PET) scan was obtained for further diagnostic clarification (Figure 1). FDG-PET scan showed diffusely decreased occipital lobe activity with sparing of the cingulate gyrus. This result suggested differentials including LBD and PCA. Rivastigmine was started for cognitive fluctuations related to possible LBD and titrated up to a dose of 4.5 mg daily. Olanzapine was tapered down to avoid the dopaminergic effects with a possible LBD diagnosis.

After the modifications to his medication regimen, the patient began acting more cooperatively and was less concerned with his paranoid thoughts. This trend continued for a few more days, including on day 15 of hospitalization when the patient reported going through his phone and not finding any evidence of fraud. The patient also began sleeping better and having a more euthymic mood and affect. On hospital day 21, the patient was discharged with a plan to follow-up with outpatient psychiatry and to receive a dopamine transporter (DAT) scan for further diagnostic clarification.

Three months after discharge, the patient received a DAT scan revealing no scintigraphic evidence for dopaminergic deficit excluding the diagnosis of LBD. Given these results, along with the patient's history, neuroimaging, cognitive testing, and clinical exam, his diagnosis was suggestive of PCA.

## 3. Discussion

In this case, the patient exhibited depressive and psychotic symptoms while not demonstrating clear memory impairment until neuropsychological testing was completed. Prior to this admission, the patient was thought to be suffering from worsening symptoms of MDD and GAD, which were diagnosed during inpatient admissions at outside facilities. However, upon admission to our hospital, the patient's psychotic symptoms were promptly recognized. The initial delay in recognition of psychotic symptoms may be attributable to the late age of onset, nonbizarre nature of the delusions, and relative preservation of cognition. The identification of these symptoms led to the initiation of antipsychotic treatment, subsequent neuroimaging, and neuropsychological testing. The medical workup pursued while the patient was admitted revealed an underlying neurodegenerative disorder. Based on the decreased activity in the bilateral occipital lobes combined with lack of dopaminergic deficit seen on DAT scan, the most likely underlying diagnosis was PCA, a variant of AD.

LBD was also considered on the differential for this patient based on his presentation which included fluctuating levels of alertness, visual hallucinations, rigidity, tremor, and a robust response to rivastigmine. Additionally, this patient showed hypometabolism in the occipital and temporoparietal cortices as well as sparing of the posterior cingulate gyrus on ^18^F-FDG-PET (Figure 1). LBD and PCA both demonstrate these patterns on ^18^F-FDG-PET, which can be utilized to differentiate from areas of hypometabolism seen in typical AD [9, 10]. While the pattern of hypometabolism is often more widespread in LBD and asymmetric in PCA [11], this patient's imaging was not widespread or asymmetric enough for these factors to weigh into the diagnosis. Ultimately, the DAT scan provided diagnostic clarification between these two diagnoses using direct dopaminergic evidence.

Both AD and PCA have a large psychiatric symptom profiles associated with them including depression, irritability, anxiety, and psychosis [12]. PCA, in particular, is often misdiagnosed as depression or anxiety in the early stages of its course [7, 8]. This delay in diagnosis may be due to early sparing of patients' memory in favor of mood and visual symptoms.

PCA is often characterized by a significant visual dimension to the presentation, correlatable to its effect on the occipital lobes. This includes “positive perceptual phenomena” such as the perception of movement of static stimuli and prolonged color after images [13]. In this patient, while his acuity and visual fields were intact, he did have visual hallucinations of dust mites moving around his room and a preoccupation with his rosacea which may have been related to positive perceptual phenomena. Furthermore, neuropsychological testing revealed impaired visuospatial construction.

This patient's symptom course lasted for 6 months including one serious SA. Despite two separate prior presentations to psychiatrists during this period and one hospitalization, the patient was assumed to have a mood disorder and was treated mainly with antidepressants. Once the patient was diagnosed with a neurodegenerative disorder, he received appropriate medication interventions and outpatient services for dementia.

Diagnosing atypical presentations of dementia is challenging due to the heterogeneous nature of symptom profiles [2, 4, 5]. This is particularly true in patients that present with primarily mood or psychotic symptoms [1, 3]. This paper demonstrates the importance of thoroughly evaluating late onset psychiatric symptoms which can often present atypically. Furthermore, we highlight the benefit of screening for neurodegenerative disorders in patients with late life affective or psychotic symptoms. This approach can ultimately lead to improving diagnostic accuracy which will then enhance overall patient care.

## Data Availability

No data are available for this study.

## References

[1] Wagner G. S., McClintock S. M., Rosenquist P. B., McCall W. V., Kahn D. A. (2011). Major depressive disorder with psychotic features may lead to misdiagnosis of dementia: a case report and review of the literature. *Journal of Psychiatric Practice*.

[2] Zapata-Restrepo L., Rivas J., Miranda C. (2021). The psychiatric misdiagnosis of behavioral variant frontotemporal dementia in a Colombian sample. *Frontiers in Neurology*.

[3] Li X., Xiong Z., Liu Y. (2020). Case report of first-episode psychotic symptoms in a patient with early-onset Alzheimer’s disease. *BMC Psychiatry*.

[4] Butler C., Zeman A. Z. J. (2005). Neurological syndromes which can be mistaken for psychiatric conditions. *Journal of Neurology, Neurosurgery & Psychiatry*.

[5] Cummings J., Ritter A., Rothenberg K. (2019). Advances in management of neuropsychiatric syndromes in neurodegenerative diseases. *Current Psychiatry Reports*.

[6] Ziegler G. C., Haarmann A., Daniels C., Herr A. (2020). The difficult diagnosis of posterior cortical atrophy in a 62-year-old woman. *Journal of Geriatric Psychiatry and Neurology*.

[7] Wolf R. C., Schönfeldt-Lecuona C. (2006). Depressive symptoms as first manifestation of posterior cortical atrophy. *American Journal of Psychiatry*.

[8] Bailey K. C., Sandlin E. K., Brickell E. E., Gardner A. (2022). Seeing though the clutter: a case report of posterior cortical atrophy and patient-centered care. *Applied Neuropsychology: Adult*.

[9] Nestor P. J., Caine D., Fryer T. D., Clarke J., Hodges J. R. (2003). The topography of metabolic deficits in posterior cortical atrophy (the visual variant of Alzheimer’s disease) with FDG-PET. *Journal of Neurology, Neurosurgery & Psychiatry*.

[10] Minoshima S., Foster N. L., Sima A. A. F., Frey K. A., Albin R. L., Kuhl D. E. (2001). Alzheimer’s disease versus dementia with Lewy bodies: cerebral metabolic distinction with autopsy confirmation. *Annals of Neurology*.

[11] Whitwell J. L., Graff-Radford J., Singh T. D. (2017). ^18^ F-FDG PET in posterior cortical atrophy and dementia with lewy bodies. *Journal of Nuclear Medicine*.

[12] Suárez-González A., Crutch S. J., Franco-Macías E., Gil-Néciga E. (2016). Neuropsychiatric symptoms in posterior cortical atrophy and Alzheimer disease. *Journal of Geriatric Psychiatry and Neurology*.

[13] Crutch S. J., Lehmann M., Schott J. M., Rabinovici G. D., Rossor M. N., Fox N. C. (2012). Posterior cortical atrophy. *The Lancet Neurology*.

